# Editorial: Aging in multiple sclerosis: from childhood to old age, in women and men

**DOI:** 10.3389/fneur.2024.1368420

**Published:** 2024-02-07

**Authors:** Alessandra Lugaresi, Vilija G. Jokubaitis, Kristen M. Krysko

**Affiliations:** ^1^IRCCS Istituto delle Scienze Neurologiche di Bologna, Bologna, Italy; ^2^Dipartimento di Scienze Biomediche e Neuromotorie, Università di Bologna, Bologna, Italy; ^3^Department of Neuroscience, School of Translational Medicine, Monash University, Melbourne, VIC, Australia; ^4^Department of Neurology, Alfred Health, Melbourne, VIC, Australia; ^5^Division of Neurology, Department of Medicine, BARLO MS Centre, St. Michael's Hospital, University of Toronto, Toronto, ON, Canada; ^6^Li Ka Shing Knowledge Institute, Toronto, ON, Canada

**Keywords:** multiple sclerosis, work, sex, pediatric, treatment, MRI, cancer

This Research Topic is dedicated to our dear friend Prof Yara Fragoso, the fourth member of our editorial team who passed away unexpectedly during the compilation of this work. Yara was a well-respected researcher and physician who specialized both in Migraine and Multiple Sclerosis. She was the president of the Brazilian Congress of Headache and Orofacial Pain at the time of her passing. She was a leader in women's health research in multiple sclerosis, and an active contributor to the international MSBase Registry, where she helped to develop a pregnancy, neonatal outcomes and women's health registry (1). We miss her deeply.

Multiple sclerosis (MS) is the most common inflammatory disease of the central nervous system and the main cause of non-traumatic disability in young adults, affecting more than 2.8 million people worldwide (2). Although it is typically diagnosed in people aged between 20–40 years, MS onset can be earlier (pediatric onset MS, POMS) or after the age of 55 (late onset MS, LOMS).

The Research Topic “*Aging in multiple sclerosis: from childhood to old age, in women and men*” consists of 12 articles providing insight into several of these aspects of MS, including aging, menopause, reproductive issues, and providing considerations on treatment, working impairment, socio-economic burden, and frailty. Assessment of the role of aging, sex hormones, diet, and infections, has enhanced our understanding in many research areas, from immunology to imaging techniques to psychology; topics further explored in the context of multiple sclerosis in this issue.

In their extension of the 2019 reviews on neurological disorders (3) and MS (4) global, regional and national burden, Qian et al. address the issue of changes reported in the incidence, mortality, disability-adjusted life years (DALYs), in people with MS all of which have increased. Whereas, age-standardized rates, calculated from the available GBD 2019 data have decreased.

With improvement of diagnostic and treatment algorithms and consequently of outcomes, the epidemiology of MS has shifted to an older than previously described population, with a peak prevalence in the 55–65 years age group. Around 50-years of age there is a consistent peak of disability accrual. Macaron et al. provide thoughtful aging related treatment considerations, taking into account confounders linked to aging, comorbidities and consequent frailty. They suggest, in the setting of aging, to monitor concomitant symptomatic treatment and to switch to safer DMTs or to de-escalate treatment through administration interval extension, depending on individual characteristics.

The impact of MS on working ability and productivity have been studied by Moccia et al. through a cross-sectional study. They have found that in MS working ability is decreased compared to matched controls, with an impact of fatigue and cognitive dysfunction, leading to lower quality of life.

The socio-economic consequences of physical and mental disability later in life have been extensively studied in Denmark, where elderly persons with MS (PwMS) face unemployment, reduced income, and increased dependence on social care (Wandall-Holm et al.).

In their narrative review Capasso et al. highlight the importance of a timely diagnosis both in POMS and LOMS, to overcome challenges encountered along the whole course of MS, including improving communication and active involvement of PwMS and caregivers.

As both mental and physical disability progress steadily in the secondary progressive phase of MS, the timely identification of patients at risk might positively impact further disease course. Tartaglia et al. suggest use of the frailty index, which correlates with the neurophysiological index and neurodegenerative rather than inflammatory processes, to predict conversion to progression.

Along this line the systematic review by Tokarska et al. highlights the use of magnetic resonance imaging (MRI) in investigating the neurobiological aging process, including physical and cognitive deterioration in PwMS, addressing questions such as (1) how does brain structure (e.g., volume, white matter microstructure) differ or change with age in PwMS? Studies show an accelerated whole brain and gray matter atrophy in PwMS; (2) Are there specific structural MRI findings in older PwMS compared to younger PwMS? This issue is still controversial, due to the lack of sufficient data available, but there is some evidence that aging may have differential effects on brain atrophy in PwMS across the lifespan as compared to normal aging; (3) Are there structural differences in the brain as a function of sex in aging PwMS? Despite the insufficient number of studies investigating this issue, the evidence to date suggests that males have greater brain atrophy than females with age, corresponding to more rapid disability accumulation and cognitive decline in males than in females; it has also been suggested that menopause may affect brain atrophy patterns in aging females with MS.

Further, the multicenter retrospective study conducted in China on 208 PwMS investigated the sex-related differences in connectivity strength and time variability within large-scale networks in relapsing remitting (RR) MS, showing alterations in connectivity strength only in male PwMS and time variability in female PwMS, suggesting that sex-related mechanisms may play an important role in the functional impairment and reorganization of cerebral activity in RRMS (Wang et al.).

With aging many PwMS present with co-morbidities which might decrease the performance of the central vein sign (CVS) in the diagnosis of MS. Lapucci et al. investigated this issue in 5,303 lesions selected for the CVS assessment in 120 MS patients stratified into 4 age groups. They found that age and migraine have a relevant impact in reducing the percentage of perivenular lesions, particularly in the deep/subcortical WM. They suggest use of the Spherical Mean Technique (SMT) diffusion model, as a helpful tool to differentiate perivenular lesions, characterized by higher inflammation, demyelination and fiber disruption, from non-perivenular lesions secondary to a different pathophysiology, especially in the deep/subcortical white matter of older patients (Lapucci et al.).

Regarding women with MS (WwMS), strategies to manage pregnancy planning have significantly changed in the last 30 years. A Delphi survey conducted in Italy led to the formulation of 21 statements in relation to optimizing “time to pregnancy”. Statements dealt with fertility considerations, treatment strategies to be adopted in case of assisted reproductive technologies and consideration for oocyte cryopreservation in women with reduced ovarian reserve, who require unpredictable time to complete diagnostic workup and achieve control of their MS (Carbone et al.).

Lorefice et al. investigated the possible role of menopause in influencing MS from clinical and neuroradiological perspectives, with special attention on brain atrophy. They found that menopause may facilitate cortical GM atrophy, probably due to a decline in the neuroprotective effects of estrogen.

The incidence of cancer in MS and the effects of treatment have not been thoroughly investigated and available results are conflicting. More specifically, there are limited data on the effect of DMTs on cervical cancer risk in WwMS. In their review Bridge et al. report the different risks associated with low, moderate and high efficacy drugs, according to different modes of action. They also take into account the possible positive effects of cervical screening programs and HPV vaccination against the barriers which preclude preventative health assessments in more advanced cases.

We hope to have provided readers with new insight in the complexities of aging with MS.

Topics still needing consideration include the impact of diagnosis on personal and sexual relationships; the role of pre-puberty and puberty in pediatric onset MS; the role of sex hormones during childbearing age (in particular hormone therapy and changes in pregnancy and postpartum); the interplay between menopause and aging; MRI techniques to investigate sexual dysfunction in male and female patients; cognition across different age groups in POMS, adult-onset MS and LOMS; immune-senescence in MS, amongst others.

## Author contributions

AL: Conceptualization, Supervision, Writing – original draft, Writing – review & editing. VJ: Writing – review & editing. KK: Writing – review & editing.

## References

[1] Jokubaitis VG Skibina O Alroughani R Altintas A Butzkueven H Eichau S. The MS base pregnancy, neonatal outcomes, and women's health registry. Ther Adv Neurol Disor. (2021) 14:1–8. 10.1177/17562864211009104 PMC8047930 33912245

[2] Mult Scler J. Multiple sclerosis under the spotlight. Lancet Neurol. (2021) 20:497. 10.1016/S1474-4422(21)00170-8 34146496

[3] Feigin VL Nichols E Alam T Bannick MS Beghi E Blake N. Global, regional, and national burden of neurological disorders, 1990-2016: a systematic analysis for the Global Burden of Disease Study 2016. Lancet Neurol. (2019) 18:459–480. 10.1016/S1474-4422(18)30499-X 30879893 PMC6459001

[4] Wallin MT Culpepper WJ Nichols E Bhutta ZA Gebrehiwot TT Hay SI. Global, regional, and national burden of multiple sclerosis 1990-2016: a systematic analysis for the Global Burden of Disease Study 2016. Lancet Neurol. (2019) 18:269–285. 10.1016/S1474-4422(18)30443-5 30679040 PMC6372756

